# Association Between HLA Genotype and Cutaneous Adverse Reactions to Antiepileptic Drugs Among Epilepsy Patients in Northwest China

**DOI:** 10.3389/fneur.2019.00001

**Published:** 2019-01-29

**Authors:** Xu Wang, Lina Chao, Xiaojing Liu, Xianrui Xu, Qing Zhang

**Affiliations:** ^1^Department of Neurology, General Hospital of Ningxia Medical University, Yinchuan, China; ^2^Department of Neurology, The First People's Hospital, Shizuishan, China; ^3^Department of Neurology, The First Hospital of Tongxiang, Tongxiang, China; ^4^Ningxia Key Laboratory of Cerebrocranial Diseases, The Incubation Base of National Key Laboratory, Yinchuan, China

**Keywords:** antiepileptic drugs, Chinese, epilepsy, HLA genotype, cutaneous adverse reaction

## Abstract

This study aimed to investigate the association between HLA genotypes and antiepileptic drug-induced cutaneous adverse reactions (AEDs-cADRs) among patients with epilepsy in Ningxia Hui Autonomous Region of Northwest China. Fifteen patients with AEDs-cADRs and 30 matched AEDs tolerant controls from anested case-control study were tested the HLA-A, HLA-B, and HLA-DRB1 genotype using the polymerase chain reaction sequence-based typing (PCR-SBT). Significant difference was not observed between AEDs-cADRs and AEDs tolerant groups in terms of HLA-A, HLA-B, and HLA-DRB1 genotype frequencies. Future studies using larger cohorts are needed to verify this observation.

## Introduction

Cutaneous adverse drug reactions (cADRs) are common adverse reactions observed in patients using antiepileptic drugs (AEDs). Studies have demonstrated that the incidence of AEDs-cADRs was about 3.61%. Also, cADRs are relatively common with the use of aromatic antiepileptic drugs (AAEDs), including carbamazepine (CBZ), phenytoin (PHT), lamotrigine (LTG), and phenobarbital (PB) (1). They manifest as ordinary maculopapular eruption (MPE), eventually leading to serious life-threatening conditions such as hypersensitivity syndrome (HSS), Steven-Johnson syndrome (SJS), or toxic epidermal necrolysis (TEN). AEDs-cADRs often lead to drug discontinuation in patients with epilepsy, resulting in the inability to control seizures. A 40% mortality rate has been reported in patients with severe cADRs (2).

Since 2004, a number of studies have suggested a strong association of HLA genotype with the occurrence of AEDs-cADRs. However, this association differs between different races and areas (3–5). In China, the majority of studies have been carried out in southern Chinese Han population (6–8). Since the seventh century, central Asians, Arabs, and Persians have migrated to China and settled to gradually form the Hui ethnicity. Some studies have suggested genetic differences between the Hui and the Han (9). Ningxia, located in Northwest China, has the largest Hui population in China. Therefore, Ningxia is an ideal state to study regional and ethnic differences. A nested case-control study was conducted in patients who were AEDs-cADRs and AEDs tolerant to determine the association between HLA genotypes and patients with AEDs-cADRs in Ningxia.

## Materials and Methods

### Study Participants

Study participants were patients diagnosed with epilepsy by the Department of Neurology in Ningxia Medical University General Hospital. The inclusion criteria were as follows: ① Ningxia resident with no history of marriages with other ethnic groups for more than three generations; ② Clear indications for AEDs treatment; ③ Have not been administered oral AEDs, and potential adverse drug reactions declared in patients or their guardians, after which signed informed consents were obtained; and ④ The initial dose and increasing dose of AEDs determined according to the “Pharmacopeia of People's Republic of China” (2010 edition). The exclusion criteria were as follows: ① Having a history of alcohol-related epilepsy; ② Having a treatable cause (such as metabolic disorders, poisoning, and infection); ③ With progressive brain or central nervous system diseases, such as encephalitis, tumors, or degenerative diseases; ④ Suffering from other diseases and the emergence of allergy during the follow-up period; and ⑤ Having to discontinue or substitute medications and not completing 12 weeks of prescribed oral AEDs.

**Figure 1:**
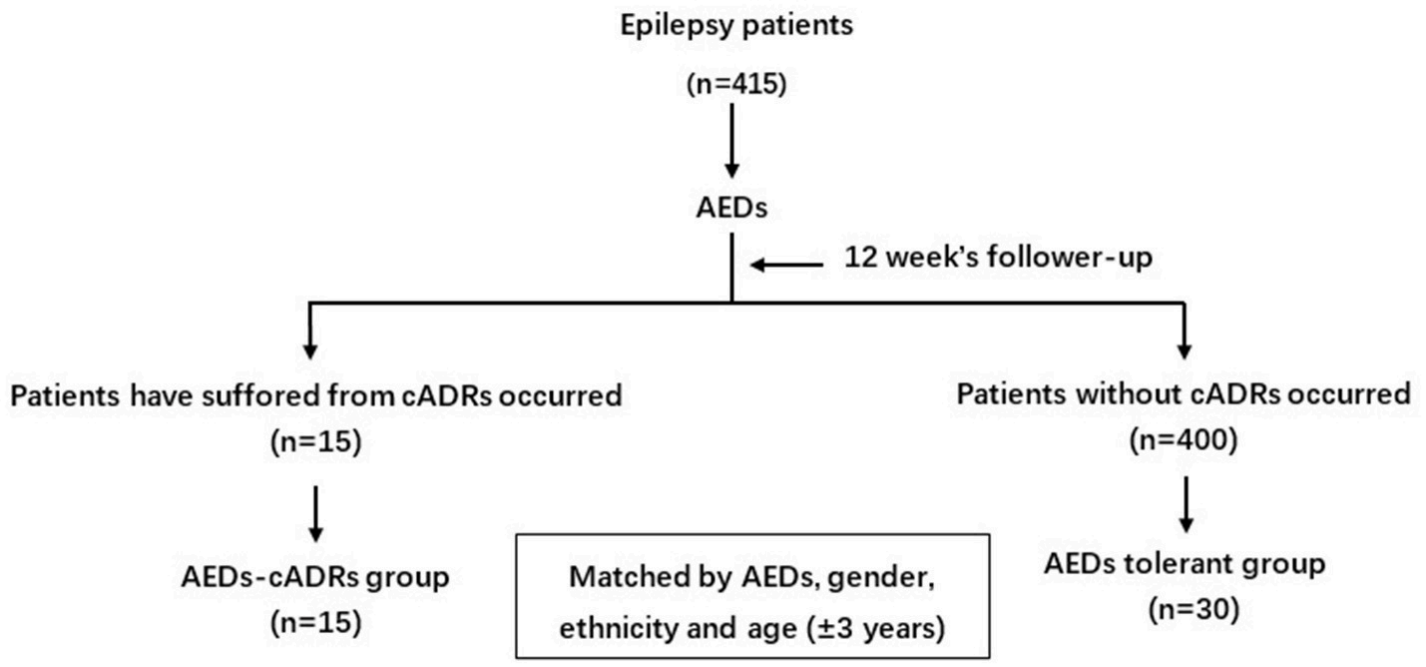
Flow chart of the nested case-control study.

Four hundred and fifteen patients were followed up bi-weekly for 12 weeks after initiating oral AEDs. The initial dosage of PHT, LTG, CBZ, and valproate (VPA) was 200, 500, 12.5, 100 mg/d, and 5 mg/kg/d, respectively. They were examined for symptoms and signs of cADRs in an epileptic clinic every 2 weeks. AEDs tolerance was defined as patients who were able to tolerate AEDs without cADRs manifestation. If cADRs manifested, the AEDs were discontinued immediately and a dermatologist was consulted to diagnose and treat the patients (Figure 1).

Two attending or one chief physician from the Department of Dermatology examined the patients. The criteria for the diagnosis and classification of cADRs were as follows: ① MPE: a rash, not involving the mucosa, no organ or system damage, and resolved after 1–2 weeks; ② HSS: in addition to skin rash, numerous viscera involvement with systemic manifestations, such as fever, arthralgia, eosinophilia, and lymphadenopathy; ③ SJS: the occurrence of skin exfoliation, involving a range of no <10% of the body area, with or without other organ or system damage; ④ TEN: the presence of skin exfoliation, involving more than 30% of the body area, with or without other organ or system damage; and ⑤ SJS/TEN: the presence of skin exfoliation, involving a range of 10–30% of the total body area. The patients were treated for skin damage based on the severity as determined by a dermatologist after cADRs diagnosis was confirmed. These patients were assigned to the AEDs-cADRs group.

Nested case-control design is the most common way to reduce the costs of exposure assessment in prospective epidemiological studies. They can also reduce the sample size through matching (10). In this study, 15 patients with epilepsy who developed cADRs were defined as the AEDs-cADRs group. For each patient with AEDs-cADRs, two patients with AEDs tolerance were selected and matched by AEDs, gender, age (±3 years), and ethnicity.

### Clinical Data Collection

A unified AEDs-cADRs epidemiological questionnaire, including demographics, underlying diseases, medication history, allergies, and seizure history, was used. The occurrence of cADRs during the 12 weeks was recorded, including the date of cADRs manifestation and other clinical manifestations involving the mucosa and subtypes.

### Ethics Statement

The General Hospital of Ningxia Medical University Ethics Committee approved the study. Also, the study was performed in compliance with the Helsinki Declaration. Access to the patient information database was granted by the General Hospital of Ningxia Medical University and approved by the ethics committee following study review. All enrolled patients agreed to have their data published and signed a written informed consent form.

### HLA-A, HLA-B, and HLA-DRB1 Genotyping

Peripheral venous blood (3 mL) from each participant was collected in anticoagulant tubes. An extraction kit (Beijing Tiangen Biotech Company, China) was used to extract genomic DNA from whole blood according to the manufacturer's protocols. HLA genotype was performed using PCR-SBT at the Beijing Boao Crystal Biotechnology Company, China. The following procedural steps were adopted: ① amplification of HLA-A, HLA-B, and HLA-DRB1 loci; ② purification and detection of the amplified products; and ③ HLA genotyping sequencing using a 3730XL ABI detector.

### Statistical Analysis

Continuous variables were expressed as mean and standard deviation (SD) and categorical variables as frequencies (%). The Pearson chi-squared test was used to compare categorical variables and the Student *t*-test to compare continuous variables. Differences in HLA genotype frequency between the groups were analyzed using the Fisher's exact test. Risk association between HLA alleles and AEDs-cADRs were presented as odds ratios (OR) and 95% confidence intervals (CI). *P*-values and 95% CIs were estimated using two-tailed tests. Data were analyzed using SPSS13.0 software.

## Results

### Characteristics of the Study Participants

The age of the 15 patients with AEDs-cARDs ranged from 14 to 60 years old, with an average age of 39.2 ± 15.4 years old. These included 10 male patients and 5 female patients with a ratio of 2:1. Twelve patients were of Han ethnicity and three of Hui ethnicity. Ten patients were with generalized epileptic seizures and five were with partial epileptic seizures. One patient had a pollen allergy history. One patient received PHT that induced cADRs, two patients received CBZ, two received LEV, one received VPA, and nine received LTG. Moreover, there were 14 patients with MPE and one Hui female patient with HSS who accepted treatment after onset. Further, five patients had cADRs after taking the initial dose of AEDs, of which one patient received CBZ, one received LEV, and three received LTG. The average time from patients taking AEDs to the occurrence of cADRs was 93.4 ± 70 days, with the longest latency period being 6 months and the shortest being 1 day (Table 1).

**Table 1:** The characteristics of AEDs-cADRs group.

**AEDs-cADRs group**	**Gender**	**Age (years)**	**Ethnicity**	**Address (city)**	**Allergy history**	**Type of AEDs**	**Daily dose**	**Time of occurrence**	**Type of cARDs**
C01	M	19	Han	Zhongning	No	PHT	300 mg	After 6 m	MPE
C02	M	14	Han	Shizuishan	No	LEV	1250 mg	After 5 d	MPE
C03	F	20	Han	Yinchuan	No	CBZ	100 mg	After 6 d	MPE
C04	M	41	Han	Yinchuan	No	CBZ	200 mg	After 43 d	MPE
C05	F	25	Han	ShiZuiShan	No	LTG	6.25 mg	After 5 d	MPE
C06	F	31	Hui	Yinchuan	YES	LTG	50 mg	After 7 d	MPE
C07	M	14	Han	ShiZuiShan	No	LEV	500 mg	After 1 m	MPE
C08	M	27	Han	ShiZuiShan	No	LTG	12.5 mg	After 2 m	MPE
C09	F	41	Hui	Pengyang	No	LTG	25 mg	After 1 m	HSS
C10	M	55	Han	Yinchuan	No	LTG	25 mg	After 2 m	MPE
C11	M	40	Hui	Yongning	No	LTG	50 mg	After 15 d	MPE
C12	M	22	Han	Jingyuan	No	LTG	25 mg	After 13 d	MPE
C13	M	60	Han	ZhongNing	No	VPA	400 mg	After 1 d	MPE
C14	F	16	Han	Guyuan	No	LTG	12.5 mg	After 10 d	MPE
C15	M	55	Han	Yinchuan	No	LTG	25 mg	After 5 d	MPE

The age of the 30 AEDs tolerant patients ranged from 12 to 65 years old, with an average age of 31.7 ± 12.4 years old. These included 19 male patients and 11 female patients at a ratio of 1.7:1. Further, 26 patients were of Han ethnicity and 4 were of Hui ethnicity. Twenty-three had generalized seizures, and seven had partial seizures. Moreover, two received PHT, four received LEV, four received CBZ, two received VPA, and eighteen received LTG (Table 2).

**Table 2:** The characteristics of AEDs tolerant group.

**AEDs tolerant group**	**Gender**	**Age (years)**	**Ethnicity**	**Address (city)**	**Type of AEDs**	**AEDs tolerance group**	**Address (city)**	**Gender**	**Age (years)**	**Ethnicity**	**Type of AEDs**
M01	M	24	Han	Qingtongxia	PHT	M16	Yongning	M	26	Han	LTG
M02	M	24	Han	Yongning	PHT	M17	Yongning	F	25	Hui	LTG
M03	M	17	Han	Jingyuan	LEV	M18	Yongning	F	25	Hui	LTG
M04	M	24	Han	Yanchi	LEV	M19	Yinchuan	M	30	Han	LTG
M05	F	27	Han	Helan	CBZ	M20	Yinchuan	M	65	Han	LTG
M06	F	25	Han	Zhongwei	CBZ	M21	Helan	M	35	Han	LTG
M07	M	45	Han	Wuzhong	CBZ	M22	Helan	M	36	Han	LTG
M08	M	39	Han	Zhongning	CBZ	M23	Shizuishan	M	17	Han	LTG
M09	F	17	Han	Yingchuan	LTG	M24	Zhongwei	M	28	Han	LTG
M10	F	22	Han	Yongning	LTG	M25	Zhongwei	M	32	Han	VPA
M11	F	27	Hui	Yongning	LTG	M26	Yinchuan	M	12	Han	VPA
M12	F	20	Hui	Yanchi	LTG	M27	Qingtongxia	F	20	Han	LTG
M13	F	23	Han	Yinchuan	LEV	M28	Helan	F	44	Han	LTG
M14	M	21	Han	Yinchuan	LEV	M29	Helan	M	23	Han	LTG
M15	M	35	Han	Helan	LTG	M30	Zhongning	M	30	Han	LTG

### Genotypes of HLA-A, HLA-B, and HLA-DRB1

In the AEDs-cADRs group, the number of HLA-A, HLA-B, and HLA-DRB1 genotypes detected was 12, 18, and 15, respectively. Higher distribution frequencies of HLA-A genotype were A^*^0207 (16.67%) and A^*^2402 (13.33%). The highest distribution frequency of HLA-B genotype was B^*^5101 (20%). The distribution frequency of A^*^0201, A^*^0206, A^*^3101, A^*^3303, B^*^3501, DRB1^*^0701, DRB1^*^0803, DRB1^*^0901, DRB1^*^1101, DRB1^*^1202, and DRB1^*^1454 was 10% (Table 3).

**Table 3:** Genotypes of HLA-A, HLA-B, and HLA-DRB1 in AEDs-cADRs group.

**AEDs-cADRs group**	**Type of AEDs**	**Type of cADRs**	**HLA sub-type**					
			HLA-A^*^		HLA-B^*^		HLA-DRB1^*^	
C01	PHT	MPE	3201	3303	3501	4403	0405	0803
C02	LEV	MPE	0207	3201	4601	5201	0901	1202
C03	CBZ	MPE	1101	2402	1505	5101	0803	1210
C04	CBZ	MPE	0201	2402	1518	5101	1101	1454
C05	LTG	MPE	0201	0207	1501	4801	0405	1405
C06	LTG	MPE	0203	0207	4601	5801	0301	1454
C07	LEV	MPE	0206	1101	1518	3501	0401	1501
C08	LTG	MPE	0206	3303	4403	5101	1302	1454
C09	LTG	HSS	2402	3101	2705	5101	0101	0701
C10	LTG	MPE	0201	2402	1302	1501	0701	1501
C11	LTG	MPE	0101	0206	1301	5701	0401	0901
C12	LTG	MPE	2901	3101	0705	5101	0803	1202
C13	VPA	MPE	3101	3303	4006	5102	1101	1101
C14	LTG	MPE	0207	1101	3501	4002	0901	1202
C15	LTG	MPE	0207	3001	1302	5101	0701	1201

In the HLA naming protocol,“^*^” separates the HLA locus (A, B and DRB1) from the serotype (32, 33 and 35), eg. HLA-A^*^ 3201.

In the AEDs tolerant group, the number of HLA-A, HLA-B, and HLA-DRB1 genotypes detected was 13, 26, and 23, respectively. Higher distribution frequencies of the HLA-A genotype were A^*^0201 (21.67%) and A^*^1101 (20%). The distribution frequency of the HLA-B genotype was lower than 10%. Higher distribution frequency of the HLA-DRB1 genotype was DRB1^*^0901 (15%), followed by DRB1^*^0301(10%) and DRB1^*^1202 (10%) (Table 4).

**Table 4:** Genotypes of HLA-A, HLA-B, and HLA-DRB1 in AEDs tolerant group.

**AEDs tolerant group**	**Type of AEDs**	**HLA sub-type**					
		HLA-A^*^		HLA-B^*^		HLA-DRB1^*^	
M01	PHT	1101	2601	0801	4001	0301	0405
M02	PHT	1101	1101	3503	5102	1101	1123
M03	LEV	1101	2402	0702	5401	0405	1501
M04	LEV	1101	1101	0702	4001	0101	0901
M05	CBZ	0201	0201	4003	6701	1302	1405
M06	CBZ	1101	3303	5201	5801	0301	0803
M07	CBZ	0302	1101	0801	1502	0301	1202
M08	CBZ	0201	3303	1501	5001	0701	1101
M09	LTG	0207	1101	0702	5101	0901	1501
M10	LTG	0201	3004	5801	5801	0410	1454
M11	LTG	1101	3001	1302	5201	0701	1502
M12	LTG	0201	3303	1301	5801	0301	1202
M13	LEV	0201	0301	3502	4101	0101	0301
M14	LEV	0101	3001	1302	3503	1103	1301
M15	LTG	0101	0201	4002	4601	1101	1202
M16	LTG	0101	0101	4001	4001	1454	1454
M17	LTG	2402	2402	3802	5101	1201	1312
M18	LTG	2402	3001	3503	5401	1101	1501
M19	LTG	0203	3303	1801	5502	1104	1602
M20	LTG	0301	3303	5201	5801	0301	1502
M21	LTG	0201	2402	1501	5101	0901	1001
M22	LTG	1101	3101	0702	4001	0101	0901
M23	LTG	0201	2601	4006	4006	0803	0901
M24	LTG	3001	3101	1302	5101	0701	1454
M25	VPA	0201	0201	4006	4601	0901	1210
M26	VPA	0201	2601	1301	5701	0701	1202
M27	LTG	0201	2402	4801	5502	0901	1405
M28	LTG	0207	2402	4006	4601	0901	0901
M29	LTG	0207	1101	4601	5502	0803	1202
M30	LTG	0207	3101	4601	5101	1202	1454

In the HLA naming protocol,“^*^” separates the HLA locus (A, B and DRB1) from the serotype (32, 33 and 35), eg. HLA-A^*^ 3201.

### Association Between HLA-A, HLA-B, and HLA-DRB1 Genotypes and AEDs-cADRs, LTG-cADRs, and AAEDs-cADRs

As shown in Table 5, OR values of 15 HLA genotypes were >1. No significant differences in HLA genotype frequencies were observed between the AEDs-cADRs and AEDs tolerant group (*P* > 0.05).

**Table 5:** Association between HLA-A, HLA-B, HLA-DRB1, and AEDs-cADRs.

**HLA type**	**Frequency**		**OR(95%CI)**	***P-*value**
	**AEDs-cADRs group (2*n* = 30)**	**AEDs tolerant group (2*n* = 60)**		
A*0203	1/30 (3.3%)	1/60 (1.7%)	2.034 (0.12~33.70)	1.00
A*0207	5/30 (16.7%)	4/60 (6.7%)	2.800 (0.69~11.32)	0.15
A*2402	4/30 (13.3%)	7/60 (11.7%)	1.165 (0.31~4.34)	1.00
A*3101	3/30 (10.0%)	3/60 (5.0%)	2.111 (0.40~11.15)	1.00
A*3303	3/30 (10.0%)	5/60 (8.3%)	1.222 (0.27~5.50)	1.00
B*1501	2/30 (6.7%)	2/60 (3.3%)	2.071 (0.28~15.48)	0.60
B*1502	0/30 (0.0%)	1/60 (1.7%)	1.017 (0.98~1.05)	1.00
B*4002	1/30 (3.3%)	1/60 (1.7%)	2.034 (0.12~33.70)	1.00
B*4801	1/30 (3.3%)	1/60 (1.7%)	2.034 (0.12~33.70)	1.00
B*5102	1/30 (3.3%)	1/60 (1.7%)	2.034 (0.12~33.70)	1.00
DRB1*0405	2/30 (6.7%)	2/60 (3.3%)	2.071 (0.28~15.48)	0.60
DRB1*0803	3/30 (10.0%)	3/60 (5.0%)	2.111 (0.40~11.15)	0.40
DRB1*1201	1/30 (3.3%)	1/60 (1.7%)	2.034 (0.12~33.70)	1.00
DRB1*1210	1/30 (3.3%)	1/60 (1.7%)	2.034 (0.12~33.70)	1.00
DRB1*1302	1/30 (3.3%)	1/60 (1.7%)	2.034 (0.12~33.70)	1.00

HLA genotyping in 9 patients with LTG-cADRs identified a total of 11 HLA-A types, 13 HLA-B types, and 13 HLA-DRB1 types. In 18 patients of LTG tolerant group, 12 HLA-A types, 17 HLA-B types, and 17 HLA-DRB1 types were identified. OR values of 5 HLA genotypes were >1. No significant differences in HLA genotype frequency were found between the LTG-cADRs and LTGtolerant group (*P* > 0.05) (Table 6).

**Table 6:** Association between HLA-A, HLA-B, HLA-DRB1, and LTG-cADRs.

**HLA type**	**Frequency**		**OR(95%CI)**	***P-*value**
	**LTG-cADRs group (2*n* = 18)**	**LTG tolerant group (2*n* = 36)**		
A*0206	4/18 (22.2%)	4/36 (11.1%)	2.28 (0.50~10.5)	0.29
A*2402	2/18 (11.1%)	2/36 (5.6%)	2.12 (0.27~16.5)	0.48
A*3101	2/18 (11.1%)	2/36 (5.6%)	2.12 (0.27~16.5)	0.48
B*5101	4/18 (22.2%)	2/36 (5.6%)	4.85 (0.80~29.6)	0.76
DRB1*3303	3/18 (16.7%)	2/36 (5.6%)	3.40 (0.51~22.5)	0.19

HLA genotyping in 12 patients with AAEDs-cADRs identified a total of 12 HLA-A types, 15 HLA-B types, and 15 HLA-DRB1 types. In 24 patients of AAEDs tolerant group, 13 HLA-A types, 23 HLA-B types, and 20 HLA-DRB1 types were identified. The OR values of 13 HLA genotypes were >1. No significant difference in HLA genotype frequency was observed between AAEDs-cADRs and AAEDs tolerant group (*P* > 0.05) (Table 7).

**Table 7:** Association between HLA-A, HLA-B, HLA-DRB1, and AAEDs-cADRs.

**HLA type**	**Frequency**		**OR(95%CI)**	***P-*value**
	**AAEDs-cADRs group (2*n* = 24)**	**AAEDs tolerant group (2*n* = 48)**		
A*0203	1/24 (4.2%)	1/48 (2.1%)	2.043 (0.12~34.16)	1.00
A*0207	4/24 (16.7%)	4/48 (8.3%)	2.00 (0.50~9.70)	0.42
B*1302	2/24 (8.3%)	2/48 (4.2%)	2.091 (0.28~15.83)	0.60
B*1501	2/24 (8.3%)	2/48 (4.2%)	2.091 (0.28~15.83)	0.60
B*4002	1/24 (4.2%)	1/48 (2.1%)	2.043 (0.12~34.16)	1.00
B*4801	1/24 (4.2%)	1/48 (2.1%)	2.043 (0.12~34.16)	1.00
B*5101	6/24 (25.0%)	5/48 (10.4%)	2.87 (0.77~10.60)	0.16
DRB1*0101	1/24 (4.2%)	1/48 (2.1%)	2.043 (0.12~34.16)	1.00
DRB1*0405	2/24 (8.3%)	1/48 (2.1%)	4.273 (0.37~49.68)	0.25
DRB1*0701	3/24 (12.5%)	3/48 (6.3%)	2.143 (0.40~11.15)	0.39
DRB1*0803	3/24 (12.5%)	3/48 (6.3%)	2.143 (0.40~11.15)	0.39
DRB1*1201	1/24 (4.2%)	1/48 (2.1%)	2.043 (0.12~34.16)	1.00
DRB1*1302	1/24 (4.2%)	1/48 (2.1%)	2.043 (0.12~34.16)	1.00

## Discussion

The occurrence of AEDs-cADRs in patients with epilepsy may be influenced by gender, age, initial AEDs dosage, incremental AEDs dosage, and adding rate, with or without a history of allergies, monotherapy or polytherapy, functional status of the liver and kidney, and genetic factors (11). Chung and colleagues reported that HLA-B^*^1502 was strongly correlated with CBZ-SJS/TEN in Taiwan Han populations (4). This result was later supported by others (3), especially for serious cADRs, with HLA susceptibility genes being the most important factor.

Different AEDs induce different cADR symptoms. In a large-scale study on 3,793 patients with epilepsy taking AEDs, the overall incidence rate of AEDs-cADRs reached 3.61%. The incidence rates of AEDs induced by cADRs were as follows: LTG (11.11%), OXC (8.92%), CBZ (3.80%), PHT (1.98%), PB (0.42%), VPA (0.57%), and LEV (1.65%) (1). About 88.41% of cADRs were induced by AAEDs of CBZ (47.56%), LTG (17.07%), OXC (9.15%), PHT(9.15%), and PB(5.49%) (12). These results suggested that AAEDs were more likely to induce cADRs in clinical practice compared with other types of AEDs. In the present study, 80% of patients with AEDs-cADRs were AAEDs-cADRs (12/15). Of these, LTG-cADRs was the most common (9/15).

Correlation studies on HLA genotypes and AEDs-cADRs have been conducted in mainland China (6–8), Taiwan (4), Hong Kong (13), Southeast Asia (14–16), Japan (17), Korea (18), Europe (19), North America (20), and other regions. The reported correlations between HLA genotypes and AEDs-cADRs have the following characteristics: ① Susceptible genes associated with AEDs-cADRs may be different among different races. For example, HLA-B^*^1502 is the susceptible gene for AEDs-SJS/TEN in Han Chinese and Southeast Asians. However, in Japan, Europe, and other parts of the world, the susceptible gene for CBZ-cADRs is HLA-A^*^3101. ② In the Han population in Southeast Asia, HLA-B^*^1502 may have a susceptibility to aromatic AEDs-SJS/TEN. ③ The incidence rate of AEDs-cADRs is relevant to the distribution rate of HLA-B^*^1502 alleles among different races. The higher the distribution rate of HLA-B^*^1502 in the race, the higher the incidence rates for AEDs-cADRs.

Ningxia, located in Northwest China, is an agglomeration of Hui ethnicities that are unlike the southern Han Chinese population genetically. Whether any HLA susceptibility genes are responsible for the occurrence of AEDs-cADRs among the northwestern population in China is not known. Therefore, the distribution rate of HLA genotypes was compared in the following groups: all patients in the AEDs-cADRs group vs. patients in the AEDs tolerant group, AAEDs-cADRs group vs. AAEDs tolerant group, and LTG-cADRs group vs. LTGtolerant group. The results suggested that the HLA-A, HLA-B, and HLA-DRB1 genotype distribution frequencies were not statistically significantly different between the two groups.

The present study had some limitations that might have impacted the outcome. First, selecting a large number of patients with AEDs-cADRs was difficult. Nine patients with LTG-cADRs were enrolled in this study. However, only one patient with CBZ-cADRs, one patient with PHT-cADRs, two patients with CBZ-cADRs, two patients with LEV-cADRs, and one patient with VPA-cADRs. Future studies focusing on large-sample populations with similar epilepsy and AEDs should be conducted. Second, the study did not adjust for age, gender, and other possible confounding variables during statistical analysis due to the small sample size. This might have had a minor impact on the results because the patients and controls were matched using a nested case–control design.

## Conclusions

The presentstudy did not find a significant association between any HLA genotypes and AEDs-cADRs in patients with epilepsy in Northwest China. Future studies using larger cohorts are needed to verify this observation.

## Author Contributions

QZ conceived this study. XW, LC, XL, and XX recruited patient samples and collected clinical data. Beijing Boao Crystal Biotechnology Company Completed HLA Genotyping. XW provided statistical analyses of the patient data and laboratory analyses.

### Conflict of Interest Statement

The authors declare that the research was conducted in the absence of any commercial or financial relationships that could be construed as a potential conflict of interest.
